# 

**Published:** 1891-01

**Authors:** 


### CONTENTS:

PAGE

ORIGINAL ARTICLES:

Comparative Pathology. By J. Bland Sutton, F. R. C. S. . . . . . . . . . . . 1
A Peculiar Monstrosity. By Daniel D. Lee, M. D. V. . . . . . . . . . . . . . 8
Roaring (*Hemiplegia Laryngis*). By Leo Breisacher, V. M. D. . . . . . . . . 9
The Screw-Worm. By. M. Francis, D. V. M. . . . . . . . . . . . . . . . . 16
Open Letter—Answer to Dr. Paquin. . . . . . . . . . . . . . . . . . . . 20
Age of the Horse, Ox, Dog and Other Domesticated Animals. By R. S. Huidekoper, M.D., Veterinarian . . . . . . . . . . . . . . . . . . 25
How much Morphine Can a Dog Stand? Can Arsenic Produce Paralysis of the Lower Jaw? . . . . . . . . . . . . . . . . . . . . . . . . . 30

EDITORIAL:

Inspection of Cattle and Sheep for Export. . . . . . . . . . . . . . . . . 31
Illinois State Veterinary Medical Association, and Iowa State Veterinary Medical Association . . . . . . . . . . . . . . . . . . . . . . . 33
United States Veterinary Medical Association. . . . . . . . . . . . . . . 33

SOCIETY PROCEEDINGS:

Proceedings of the Association for the Study of Comparative Psychology, in Connection with the Faculty of Comparative Medicine and Veterinary Science, McGill University, Montreal, for the year 1889-90 . . . . 34
Keystone Veterinary Medical Association. . . . . . . . . . . . . . . . . 40
Long Island Veterinary Society. . . . . . . . . . . . . . . . . . . . . 41
Massachusetts Veterinary Association. . . . . . . . . . . . . . . . . . 44

VETERINARY COLLEGE NOTES:

Ontario Veterinary College. . . . . . . . . . . . . . . . . . . . . . . 46

REVIEW DEPARTMENT:

Are the Effects of Use and Disuse Inherited? An Examination of the View Held by Spencer and Darwin. By William Platt Ball. . . . . . . . . 40
The Physicians' Visiting List for 1891. . . . . . . . . . . . . . . . . . 57
Books and Pamphlets Received. . . . . . . . . . . . . . . . . . . . . 58

